# Supplementary material for: Reverse Engineering of the Pediatric Sepsis Regulatory Network and Identification of Master Regulators
Source: Biomedicines. 2021 Sep 23;9(10):1297. doi: 10.3390/biomedicines9101297 (PMC8533457; doi:10.3390/biomedicines9101297)
Supplement: Supplementary file 1 [file biomedicines-09-01297-s001.zip › Supplementary Figure S2.pdf]

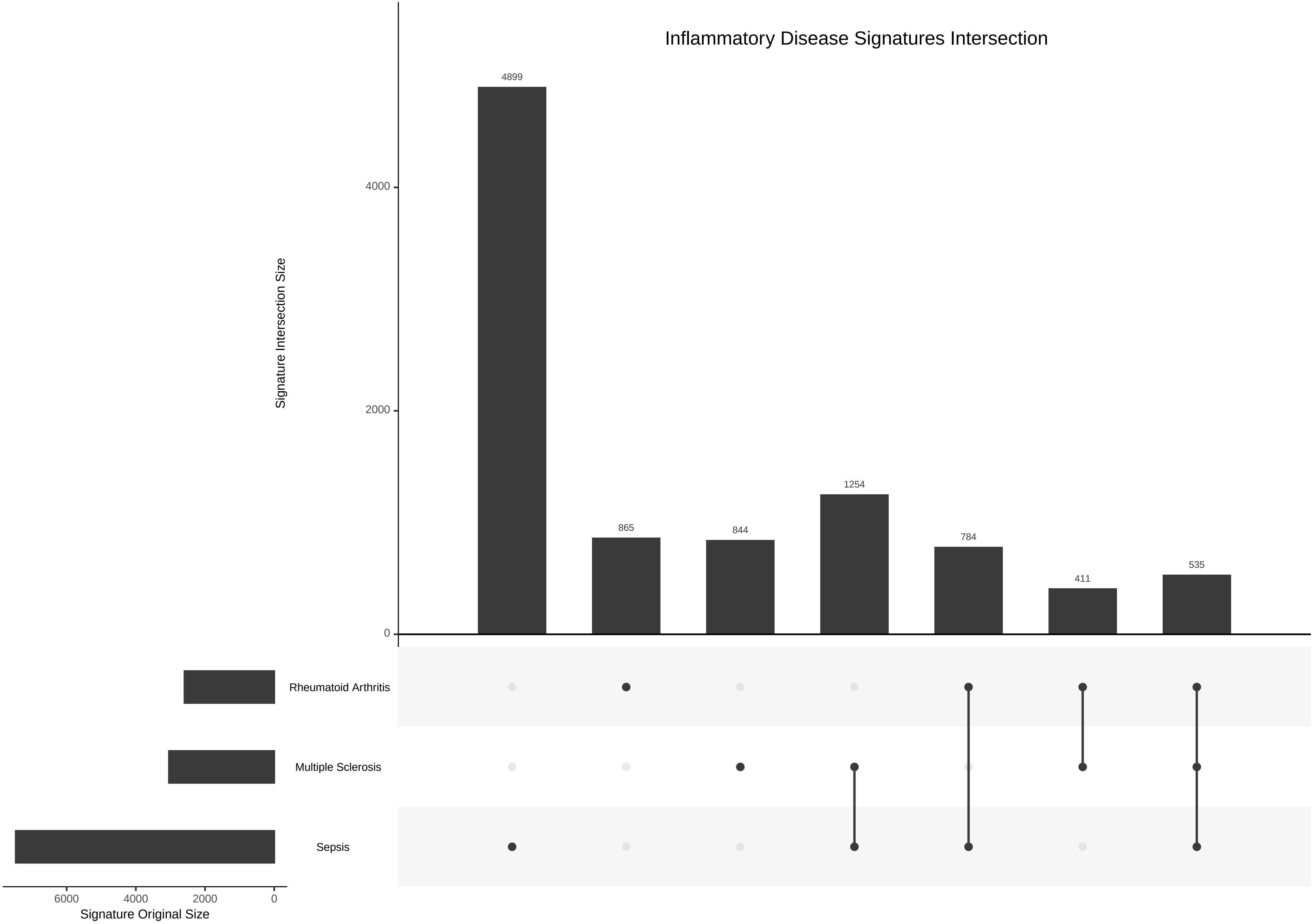
Inflammatory Disease Signatures Intersection
Signature Intersection Size
0
2000
4000
4899
865
844
1254
784
411
535
Rheumatoid Arthritis
Multiple Sclerosis
Sepsis
6000
4000
2000
0
Signature Original Size
