# Supplementary material for: Reverse Engineering of the Pediatric Sepsis Regulatory Network and Identification of Master Regulators
Source: Biomedicines. 2021 Sep 23;9(10):1297. doi: 10.3390/biomedicines9101297 (PMC8533457; doi:10.3390/biomedicines9101297)
Supplement: Supplementary file 1 [file biomedicines-09-01297-s001.zip › Supplementary Material.pdf]

Online Supplementary Material

Reverse Engineering of Pediatric Sepsis Regulatory Network and Identification of Master Regulators

Raffael Azevedo de Carvalho Oliveira [1], Danilo Oliveira Imparato [1], Vítor Gabriel Saldanha Fernandes [1], João Vitor Ferreira Cavalcante [1], Ricardo D'Oliveira Albanus [2] and Rodrigo Juliani Siqueira Dalmolin [1,3,*]

[1] Bioinformatics Multidisciplinary Environment–BioME, Universidade Federal do Rio Grande do Norte, Instituto Metrópole Digital, Natal 59078-400, Brazil; raffael.azevedo@gmail.com (R.A.d.C.O.); xdanilo@ufrn.edu.br (D.O.I.); vitor.saldanha.095@ufrn.edu.br (V.G.S.F.); jvfecav@gmail.com (J.V.F.C.)

[2] Department of Computational Medicine & Bioinformatics, University of Michigan, Ann Arbor, ML 48109, US; albanus@umich.edu (R.D.A.)

[3] Department of Biochemistry–DBQ–CB, Federal University of Rio Grande do Norte, Natal 59064-741, Brazil

* Correspondence: rodrigo.dalmolin@imd.ufrn.br

SUPPLEMENTARY MATERIAL - RESULTS AND DISCUSSION

1. Covariate selection

In order to identify sources of expression variability in the metadata, we selected three variables present in the GSE4607 dataset: Steroid, which includes information about corticosteroid treatment, Organism, which identifies the organism responsible for the condition, and Infect_site, that provides the original site of infection. Our approach to measure the importance of each of these covariates started by performing a principal component analysis (PCA) in the expression matrix of dataset GSE4607. 5 principal components (PCs) were chosen, which together explain 44.65% of gene expression variation. The relationship between the covariates and these PCs was determined through a Spearman correlation, where correlations with p-values <= 0.05 were selected. The cumulative variance of significantly correlated components for each covariate is shown on Supplementary Figure S4.

From the covariate selection, we were able to gather that steroid treatment showed the highest cumulative variance among the significantly correlated components, this indicates that steroid treatment contributes greatly to expression variability in septic shock patients, something that is explained by their mechanism of action, which is intrinsically linked to the expression of anti-inflammatory proteins and therefore associated with patient outcome.

SUPPLEMENTARY MATERIAL - FIGURES

Supplementary Figure S1: Detailed steps of filtering master regulators, in order to get to the final set of 15 MRs.

Supplementary Figure S2: Intersection between all gene signatures used in this work.

Supplementary Figure S3: Cumulative variance of significantly correlated components for each covariate selected in the dataset GSE4607.

SUPPLEMENTARY MATERIAL - TABLES

Supplementary Table S1: List of all 233 obtained from Sepsis 1 dataset. This table shows information regarding each regulon using data from GSE13904 (Sepsis 1): the number of genes in the universe, size of the regulon, total genes in the gene signature, the expected number of hits, the actual observed hits, and a p-value (along with the adjusted by FDR) for this comparison.

Supplementary Table S2: List of all 215 obtained from Sepsis 2 dataset. This table shows information regarding each regulon using data from GSE4607 (Sepsis 2): the number of genes in the universe, size of the regulon, total genes in the gene signature, the expected number of hits, the actual observed hits, and a p-value (along with the adjusted by FDR) for this comparison.

Supplementary Table S3: List of the 179 MRs common to both Sepsis 1 and Sepsis 2 networks. This table shows information regarding each regulon using data from GSE13904 (Sepsis 1): the number of genes in the universe, size of the regulon, total genes in the gene signature, the expected number of hits, the actual observed hits, and a p-value (along with the adjusted by FDR) for this comparison.

Supplementary Table S4: List of the 179 MRs common to both Sepsis 1 and Sepsis 2 networks. This table shows information regarding each regulon using data from GSE4607 (Sepsis 2): the number of genes in the universe, size of the regulon, total genes in the gene signature, the expected number of hits, the actual observed hits, and a p-value (along with the adjusted by FDR) for this comparison.

Supplementary Table S5: List of the 50 MRs present on each cluster, common to both Sepsis 1 and Sepsis 2 networks. This table shows information regarding each regulon using data from GSE13904 (Sepsis 1): the number of genes in the universe, size of the regulon, total genes in the gene signature, the expected number of hits, the observed hits, and a p-value (along with the adjusted by FDR) for this comparison. The last column indicates whether regulon belongs to cluster A or B.

Supplementary Table S6: List of the 50 MRs present on each cluster, common to both Sepsis 1 and Sepsis 2 networks. This table shows information regarding each regulon using data from GSE4607 (Sepsis 2): the number of genes in the universe, size of the regulon, total genes in the gene signature, the expected number of hits, the observed hits, and a p-value (along with the adjusted by FDR) for this comparison. The last column indicates whether regulon belongs to cluster A or B.

Supplementary Table S7: Final master regulator list of GSE13904 network. This table shows information regarding each regulon: the number of genes in the universe, size of the regulon, total genes in the gene signature, the expected number of hits, the actual observed hits, and a p-value (along with the adjusted by FDR) for this comparison.

Supplementary Table S8: Final master regulator list of GSE4607 network. This table shows information regarding each regulon: the number of genes in the universe, size of the regulon, total genes in the gene signature, the expected number of hits, the actual observed hits, and a p-value (along with the adjusted by FDR) for this comparison.

Supplementary Table S9: Reference list of each of the 15 final MR, displaying the gene name, other possible aliases that gene could have, and protein name.

Supplementary Figure S1

Master Regulators filtering

Step 1. MRA of two sepsis networks

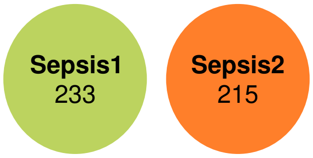

Step 2. Intersection between both networks

179 Sepsis MRs

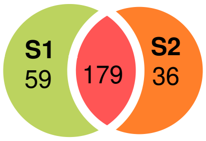

Step 3. Subset MRs by presence in sepsis network clusters

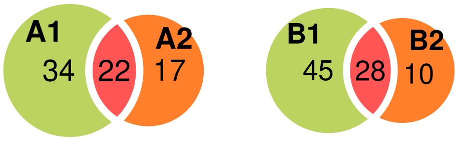

Step 4. 50 common MRs in each cluster (A and B) in both networks (Sepsis 1 and 2)

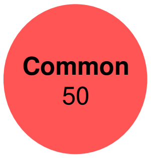

Step 5. Addition of sepsis MRs obtained from RA and MS signatures followed by intersection exclusion

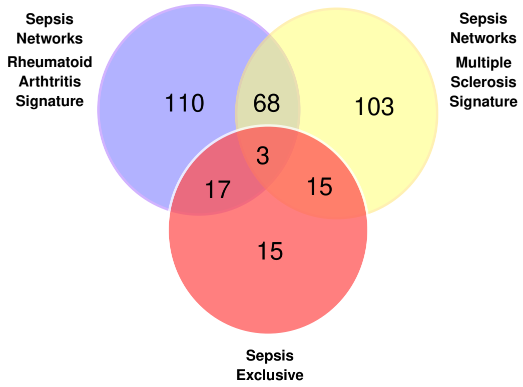

Step 6. Subset and get unique sepsis-related Master Regulators

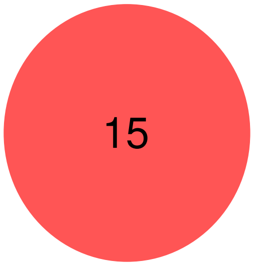

Specific MRs After Filtering

Supplementary Figure S2

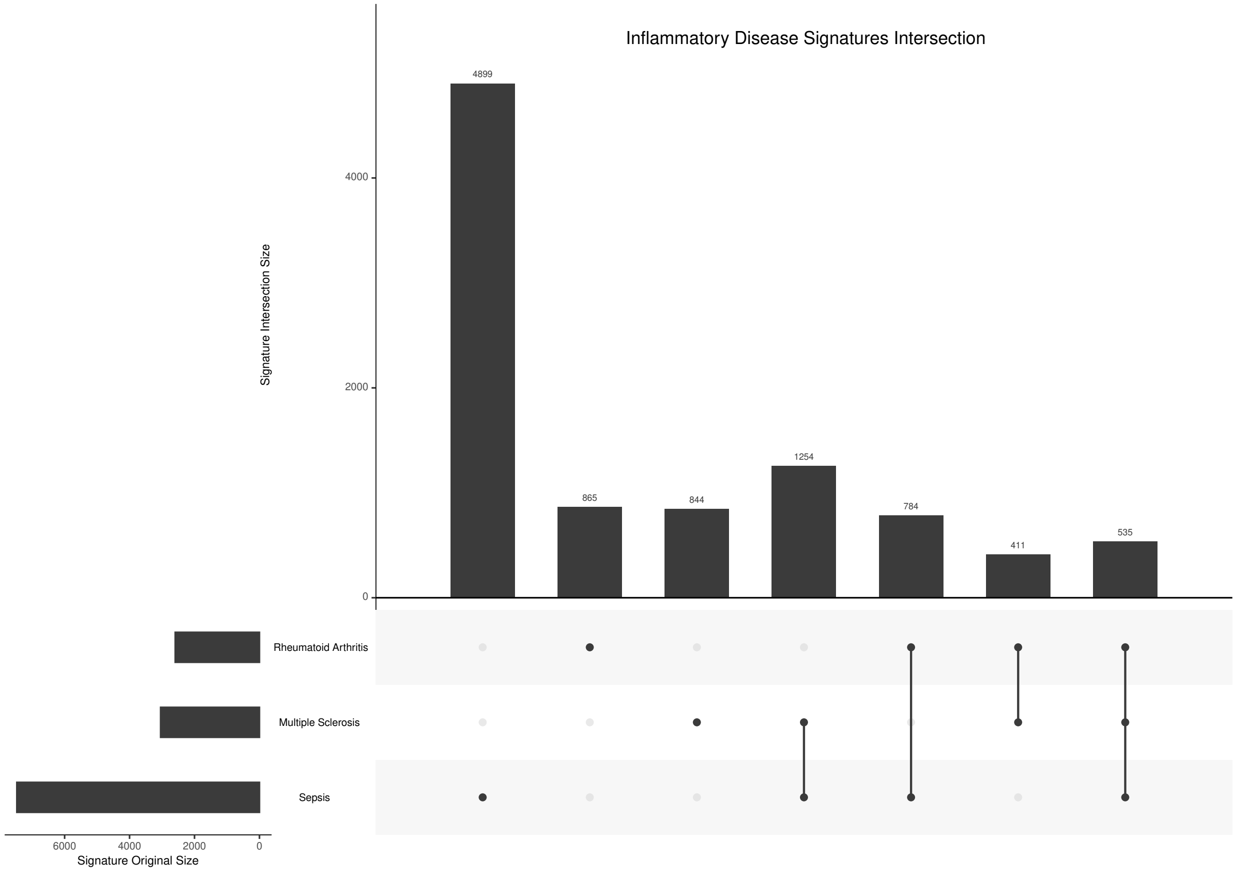

Supplementary Figure S3

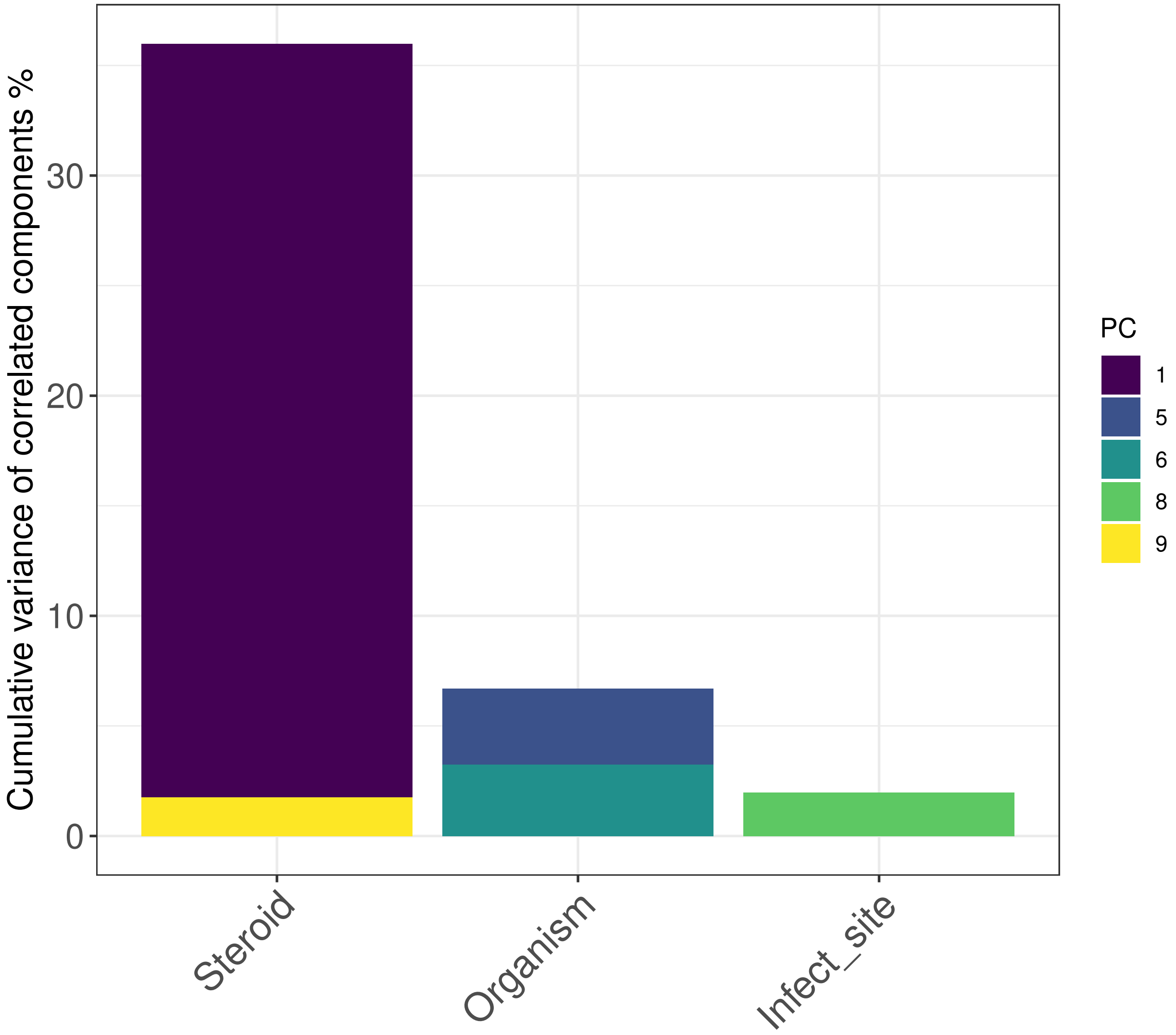

Table S1

Affymetrix probe ID	Regulon	Pvalue	Adjusted.Pvalue
218767_at	REXO4	0	0
206142_at	ZNF135	5,70E-302	2,00E-299
200878_at	EPAS1	2,50E-260	5,80E-258
214482_at	ZBTB25	1,10E-218	1,80E-216
207704_s_at	GAS7	1,10E-213	1,50E-211
231940_at	ZNF529	1,30E-196	1,50E-194
209211_at	KLF5	1,50E-195	1,50E-193
203585_at	ZNF185	1,10E-187	9,40E-186
209930_s_at	NFE2	6,60E-187	5,10E-185
212501_at	CEBPB	1,80E-185	1,30E-183
205254_x_at	TCF7	2,30E-182	1,50E-180
209782_s_at	DBP	8,60E-171	5,00E-169
203394_s_at	HES1	5,70E-169	3,00E-167
214523_at	CEBPE	3,20E-162	1,60E-160
227150_at	MTF1	1,30E-158	5,90E-157
203574_at	NFIL3	2,60E-156	1,10E-154
204970_s_at	MAFG	1,70E-153	6,80E-152
230575_at	MSRB2	2,30E-151	8,70E-150
204327_s_at	ZNF202	4,20E-147	1,50E-145
214746_s_at	ZNF467	2,40E-146	8,40E-145
209602_s_at	GATA3	7,40E-144	2,40E-142
215285_s_at	PHTF1	1,80E-140	5,80E-139
205101_at	CIITA	2,00E-135	6,10E-134
207443_at	NR2E1	5,20E-132	1,50E-130
208510_s_at	PPARG	1,30E-131	3,60E-130
218284_at	SMAD3	1,10E-125	2,80E-124
205026_at	STAT5B	1,60E-123	4,10E-122
205883_at	ZBTB16	4,00E-117	9,90E-116
218184_at	TULP4	4,40E-113	1,00E-111
211721_s_at	ZNF551	8,50E-113	2,00E-111
206838_at	TBX19	6,10E-111	1,40E-109
228099_at	ZNF550	2,20E-109	4,70E-108
206118_at	STAT4	5,50E-107	1,20E-105
205312_at	SPI1	1,90E-106	3,90E-105
218880_at	FOSL2	1,30E-105	2,60E-104
209538_at	ZNF32	5,40E-103	1,00E-101
206965_at	KLF12	8,10E-102	1,50E-100
235567_at	RORA	6,90E-98	1,20E-96
205453_at	HOXB2	1,30E-95	2,30E-94
212892_at	ZNF282	2,50E-94	4,40E-93
216350_s_at	ZNF10	7,90E-93	1,30E-91
220055_at	ZNF287	4,70E-92	7,80E-91
213269_at	ZNF248	3,50E-90	5,70E-89
204432_at	SOX12	4,30E-90	6,80E-89
216305_s_at	GCFC2	4,80E-86	7,40E-85
205259_at	NR3C2	1,60E-84	2,40E-83
226327_at	ZNF507	5,20E-83	7,70E-82
1558586_at	ZNF33B	2,70E-79	3,90E-78
206877_at	MXD1	1,30E-78	1,90E-77
219765_at	ZNF329	1,40E-74	2,00E-73
219548_at	ZNF16	3,00E-74	4,00E-73
201473_at	JUNB	4,50E-74	5,90E-73

200939_s_at	RERE	3,10E-73	4,00E-72
218437_s_at	LZTFL1	4,20E-73	5,40E-72
226872_at	RFX2	2,20E-72	2,70E-71
226102_at	ZKSCAN8	1,50E-70	1,80E-69
242915_at	ZNF682	5,70E-70	7,00E-69
207417_s_at	ZNF559-ZNF177	1,80E-69	2,10E-68
204562_at	IRF4	1,90E-69	2,20E-68
209200_at	MEF2C	3,80E-68	4,40E-67
242919_at	ZNF253	4,10E-68	4,60E-67
219228_at	ZNF331	5,00E-67	5,50E-66
201329_s_at	ETS2	2,90E-66	3,20E-65
209657_s_at	HSF2	4,90E-66	5,30E-65
206053_at	ZNF510	1,50E-64	1,60E-63
208808_s_at	HMGB2	2,30E-64	2,40E-63
205004_at	NKRF	2,60E-63	2,60E-62
201746_at	TP53	2,50E-62	2,60E-61
1555420_a_at	KLF7	9,30E-62	9,30E-61
220350_at	ZNF235	2,70E-59	2,70E-58
218735_s_at	ZNF544	4,80E-59	4,70E-58
236128_at	ZNF91	7,70E-58	7,40E-57
202963_at	RFX5	1,90E-55	1,80E-54
217627_at	ZNF573	2,30E-55	2,10E-54
208328_s_at	MEF2A	2,70E-52	2,50E-51
204039_at	CEBPA	6,10E-52	5,50E-51
220684_at	TBX21	9,80E-51	8,80E-50
202957_at	HCLS1	1,90E-49	1,70E-48
236471_at	NFE2L3	2,00E-48	1,80E-47
241365_at	SATB1	5,80E-45	5,00E-44
242953_at	ZNF234	2,50E-42	2,10E-41
215990_s_at	BCL6	4,60E-42	3,90E-41
1566324_a_at	MAF	5,70E-39	4,70E-38
227496_at	NR6A1	1,10E-38	8,90E-38
203693_s_at	E2F3	1,20E-38	9,70E-38
217909_s_at	MLX	1,30E-38	1,00E-37
202426_s_at	RXRA	2,20E-38	1,80E-37
200989_at	HIF1A	2,00E-37	1,60E-36
1554411_at	CTNNB1	6,50E-37	5,00E-36
201483_s_at	SUPT4H1	1,10E-36	8,40E-36
210062_s_at	ZNF589	1,70E-36	1,30E-35
227132_at	ZNF706	7,30E-36	5,50E-35
211097_s_at	PBX2	1,10E-35	8,40E-35
1554465_s_at	KRBOX4	7,30E-34	5,40E-33
218149_s_at	ZNF395	7,30E-33	5,30E-32
211064_at	ZNF493	1,20E-32	8,80E-32
219854_at	ZNF14	1,70E-32	1,20E-31
227045_at	ZNF614	8,90E-31	6,30E-30
204255_s_at	VDR	1,70E-30	1,20E-29
207128_s_at	ZNF223	2,00E-30	1,40E-29
228466_at	GABPB2	2,50E-30	1,70E-29
207068_at	ZFP37	3,70E-30	2,50E-29
229431_at	RFXAP	6,10E-30	4,10E-29
209189_at	FOS	5,20E-29	3,50E-28
229228_at	CREB5	7,50E-29	5,00E-28

206182_at	ZNF134	8,20E-29	5,30E-28
229913_at	C7orf61	4,00E-28	2,60E-27
225021_at	ZNF532	9,80E-28	6,30E-27
1555355_a_at	ETS1	1,40E-27	8,80E-27
219171_s_at	ZNF236	2,20E-27	1,40E-26
227198_at	AFF3	2,30E-27	1,40E-26
205865_at	ARID3A	8,10E-27	5,00E-26
211789_s_at	MLXIP	2,20E-26	1,40E-25
209798_at	NPAT	6,30E-26	3,80E-25
201507_at	PFDN1	2,00E-25	1,20E-24
1554171_at	ZMYM3	5,10E-25	3,00E-24
218006_s_at	ZNF22	2,20E-24	1,30E-23
219849_at	ZNF671	2,80E-24	1,60E-23
204194_at	BACH1	1,70E-23	9,70E-23
217185_s_at	ZPR1	2,10E-23	1,20E-22
218559_s_at	MAFB	8,30E-23	4,80E-22
218724_s_at	TGIF2	1,10E-22	6,40E-22
205287_s_at	TFAP2C	2,10E-22	1,20E-21
41037_at	TEAD4	3,00E-22	1,70E-21
208270_s_at	RNPEP	3,20E-22	1,80E-21
215933_s_at	HHEX	6,60E-22	3,60E-21
214901_at	ZNF8	1,80E-21	1,00E-20
208960_s_at	KLF6	4,60E-21	2,50E-20
212079_s_at	KMT2A	1,60E-20	8,50E-20
200050_at	ZNF146	1,30E-19	7,10E-19
224718_at	YY1	1,40E-19	7,40E-19
224806_at	TRIM25	3,50E-19	1,80E-18
235122_at	HIVEP3	4,00E-19	2,10E-18
218215_s_at	NR1H2	4,30E-19	2,20E-18
205619_s_at	MEOX1	5,90E-19	3,00E-18
205205_at	RELB	3,50E-18	1,80E-17
202758_s_at	RFXANK	5,70E-18	2,90E-17
210818_s_at	BACH1	1,10E-17	5,80E-17
204791_at	NR2C1	1,30E-17	6,40E-17
214690_at	TAF1B	1,90E-17	9,40E-17
37254_at	ZNF133	1,10E-16	5,60E-16
1554311_a_at	SUPT6H	2,70E-16	1,30E-15
203554_x_at	PTTG1	3,30E-16	1,60E-15
205658_s_at	SNAPC4	3,60E-16	1,70E-15
207120_at	ZNF667	2,30E-15	1,10E-14
226978_at	PPARA	6,00E-15	2,90E-14
204045_at	TCEAL3	7,30E-15	3,40E-14
206219_s_at	VAV1	1,50E-14	6,90E-14
1557322_at	ZNF230	6,10E-14	2,80E-13
218793_s_at	SCML1	6,30E-14	2,90E-13
207753_at	ZNF304	1,00E-13	4,70E-13
215111_s_at	TSC22D1	1,50E-13	7,00E-13
201332_s_at	STAT6	1,50E-13	7,00E-13
236265_at	SP4	4,00E-13	1,80E-12
204057_at	IRF8	1,40E-12	6,40E-12
200758_s_at	NFE2L1	1,60E-12	7,30E-12
201417_at	SOX4	1,80E-12	8,10E-12
1555325_s_at	ZNF26	5,30E-12	2,30E-11

221123_x_at	ZNF395	3,60E-11	1,50E-10
232652_x_at	SCAND1	1,90E-10	8,20E-10
209587_at	PITX1	2,60E-10	1,10E-09
218401_s_at	ZNF281	2,80E-10	1,20E-09
1565347_s_at	TFE3	4,20E-10	1,80E-09
202580_x_at	FOXM1	5,60E-10	2,30E-09
217294_s_at	ENO1	6,00E-10	2,50E-09
201218_at	CTBP2	2,20E-09	9,20E-09
218490_s_at	ZNF302	3,00E-09	1,20E-08
206931_at	ZNF141	3,10E-09	1,30E-08
209989_at	ZNF268	8,70E-09	3,60E-08
213541_s_at	ERG	1,10E-08	4,60E-08
207980_s_at	CITED2	2,40E-08	9,50E-08
203258_at	DRAP1	3,00E-08	1,20E-07
207001_x_at	TSC22D3	3,90E-08	1,60E-07
229010_at	CBL	5,60E-08	2,20E-07
201466_s_at	JUN	6,40E-08	2,50E-07
232555_at	CREB5	8,20E-08	3,20E-07
205170_at	STAT2	1,10E-07	4,30E-07
203275_at	IRF2	1,70E-07	6,70E-07
220760_x_at	ZNF665	2,10E-07	8,00E-07
210252_s_at	MADD	4,50E-07	1,70E-06
235521_at	HOXA3	5,20E-07	2,00E-06
200990_at	TRIM28	1,40E-06	5,20E-06
205881_at	ZNF74	1,50E-06	5,60E-06
218486_at	KLF11	1,60E-06	5,90E-06
218524_at	E4F1	2,20E-06	8,20E-06
31845_at	ELF4	3,30E-06	1,20E-05
209454_s_at	TEAD3	3,50E-06	1,30E-05
204275_at	CAPN15	7,00E-06	2,60E-05
50221_at	TFEB	7,40E-06	2,70E-05
205514_at	ZNF415	7,70E-06	2,80E-05
207006_s_at	CCDC106	8,70E-06	3,10E-05
227067_x_at	NBPF14	1,10E-05	3,80E-05
213452_at	ZNF184	1,10E-05	4,00E-05
203985_at	ZNF212	5,00E-05	0,00018
218152_at	HMG20A	5,10E-05	0,00018
232231_at	RUNX2	7,30E-05	0,00026
228092_at	CREM	7,90E-05	0,00028
206583_at	KRBOX4	0,00014	0,00051
207513_s_at	ZNF189	0,00016	0,00054
204798_at	MYB	0,0002	0,0007
216004_s_at	PKNOX1	0,00021	0,00071
208330_at	ALX4	0,00026	0,00088
210756_s_at	NOTCH2	0,00026	0,00089
219540_at	ZNF267	0,00028	0,00094
203543_s_at	KLF9	0,00032	0,0011
205372_at	PLAG1	0,00037	0,0012
213150_at	HOXA10	0,00043	0,0014
233461_x_at	ZNF226	0,00045	0,0015
AFFX-HUMISGF3A/M97935_5_at	STAT1	0,00047	0,0015
220748_s_at	ZNF580	0,00075	0,0025
207002_s_at	PLAGL1	0,00087	0,0029

238614_x_at	ZNF430	0,0013	0,0044
208056_s_at	CBFA2T3	0,0015	0,0047
209710_at	GATA2	0,0015	0,0047
1565358_at	RARA	0,0017	0,0055
213639_s_at	ZNF500	0,0023	0,0073
218495_at	UXT	0,0036	11
204481_at	BRPF1	0,0037	12
212641_at	HIVEP2	0,0038	12
200641_s_at	YWHAZP3	4	12
240557_at	TSC22D2	0,0049	15
208443_x_at	SHOX2	0,0056	18
203601_s_at	ZBTB17	0,0063	19
231927_at	ATF6	0,0068	21
213916_at	ZNF625-ZNF20	7	22
203132_at	RB1	0,0072	22
202925_s_at	PLAGL2	0,0082	25
36711_at	MAFF	0,0083	25
217547_x_at	ZNF675	0,0085	26
217861_s_at	PREB	0,0089	27
209088_s_at	UBN1	0,01	0,03
209944_at	ZNF410	12	35
209289_at	NFIB	13	39

Table S2

Affymetrix probe ID	Regulon	Pvalue	Adjusted.Pvalue
214482_at	ZBTB25	1,10E-237	7,90E-235
206142_at	ZNF135	1,40E-197	4,70E-195
231940_at	ZNF529	1,60E-177	3,60E-175
209930_s_at	NFE2	2,10E-167	3,60E-165
211721_s_at	ZNF551	6,80E-164	9,40E-162
204327_s_at	ZNF202	7,90E-161	9,10E-159
209602_s_at	GATA3	1,40E-154	1,40E-152
204970_s_at	MAFG	2,30E-154	2,00E-152
200878_at	EPAS1	2,60E-150	2,00E-148
227150_at	MTF1	5,00E-147	3,40E-145
213269_at	ZNF248	4,90E-138	3,10E-136
207704_s_at	GAS7	9,30E-135	5,30E-133
203574_at	NFIL3	2,60E-133	1,40E-131
212501_at	CEBPB	3,10E-120	1,50E-118
214523_at	CEBPE	1,60E-115	7,40E-114
206965_at	KLF12	4,20E-115	1,80E-113
209211_at	KLF5	6,00E-114	2,40E-112
204432_at	SOX12	3,70E-113	1,40E-111
1558586_at	ZNF33B	1,40E-107	5,10E-106
215285_s_at	PHTF1	2,40E-103	8,20E-102
226682_at	RORA	1,50E-102	5,00E-101
1555420_a_at	KLF7	2,40E-102	7,60E-101
214746_s_at	ZNF467	5,40E-102	1,60E-100
226102_at	ZKSCAN8	6,40E-101	1,80E-99
218184_at	TULP4	1,90E-95	5,10E-94
205026_at	STAT5B	1,50E-90	4,00E-89
218767_at	REXO4	4,20E-89	1,10E-87
208510_s_at	PPARG	1,80E-88	4,50E-87
209782_s_at	DBP	8,20E-87	1,90E-85
219765_at	ZNF329	3,10E-84	7,10E-83
205004_at	NKRF	1,40E-82	3,00E-81
217627_at	ZNF573	9,40E-78	2,00E-76
242919_at	ZNF253	1,00E-76	2,10E-75
220350_at	ZNF235	5,20E-76	1,10E-74
216305_s_at	GCFC2	2,80E-74	5,50E-73
218880_at	FOSL2	1,10E-69	2,00E-68
202963_at	RFX5	9,00E-69	1,70E-67
220684_at	TBX21	9,30E-69	1,70E-67
201329_s_at	ETS2	2,60E-65	4,60E-64
228099_at	ZNF550	2,70E-65	4,60E-64
218284_at	SMAD3	6,70E-64	1,10E-62
206877_at	MXD1	1,00E-62	1,70E-61
209538_at	ZNF32	2,40E-62	3,80E-61
215990_s_at	BCL6	8,70E-62	1,40E-60
201746_at	TP53	3,00E-59	4,60E-58
205453_at	HOXB2	7,90E-59	1,20E-57
212079_s_at	KMT2A	1,60E-58	2,30E-57
204198_s_at	RUNX3	5,40E-58	7,80E-57
219228_at	ZNF331	4,10E-56	5,70E-55
205312_at	SPI1	1,00E-55	1,40E-54
241365_at	SATB1	8,80E-55	1,20E-53
202431_s_at	MYC	7,70E-53	1,00E-51

204791_at	NR2C1	1,30E-50	1,70E-49
227132_at	ZNF706	1,10E-49	1,40E-48
206118_at	STAT4	1,30E-49	1,60E-48
228466_at	GABPB2	1,40E-49	1,70E-48
229431_at	RFXAP	2,90E-48	3,60E-47
205101_at	CIITA	3,70E-48	4,30E-47
226872_at	RFX2	7,70E-47	9,00E-46
218149_s_at	ZNF395	1,60E-46	1,80E-45
205865_at	ARID3A	1,70E-46	1,90E-45
219269_at	HMBOX1	4,20E-45	4,60E-44
209657_s_at	HSF2	9,00E-45	9,80E-44
203957_at	E2F6	3,90E-44	4,20E-43
209200_at	MEF2C	7,10E-44	7,50E-43
203693_s_at	E2F3	1,90E-41	2,00E-40
229228_at	CREB5	2,20E-41	2,20E-40
204194_at	BACH1	3,10E-41	3,10E-40
204039_at	CEBPA	1,80E-40	1,80E-39
211789_s_at	MLXIP	5,40E-40	5,30E-39
201473_at	JUNB	1,30E-38	1,30E-37
207753_at	ZNF304	1,70E-38	1,70E-37
206074_s_at	HMGA1	8,60E-38	8,10E-37
210062_s_at	ZNF589	1,60E-37	1,50E-36
218735_s_at	ZNF544	5,50E-37	5,00E-36
207128_s_at	ZNF223	9,30E-37	8,40E-36
219854_at	ZNF14	1,10E-36	1,00E-35
227045_at	ZNF614	1,20E-36	1,00E-35
202957_at	HCLS1	4,70E-36	4,10E-35
235122_at	HIVEP3	5,00E-36	4,30E-35
227198_at	AFF3	2,90E-35	2,50E-34
203394_s_at	HES1	1,20E-33	1,00E-32
205259_at	NR3C2	1,40E-33	1,20E-32
216350_s_at	ZNF10	1,50E-33	1,20E-32
1555355_a_at	ETS1	2,10E-33	1,70E-32
218006_s_at	ZNF22	6,70E-33	5,30E-32
209798_at	NPAT	1,20E-32	9,40E-32
219123_at	ZNF232	1,30E-32	1,10E-31
208808_s_at	HMGB2	1,60E-31	1,20E-30
200989_at	HIF1A	2,80E-31	2,10E-30
204045_at	TCEAL3	8,60E-31	6,50E-30
236128_at	ZNF91	1,60E-30	1,20E-29
212892_at	ZNF282	1,70E-30	1,30E-29
206182_at	ZNF134	1,80E-30	1,30E-29
200050_at	ZNF146	1,40E-29	1,00E-28
218401_s_at	ZNF281	1,20E-28	8,30E-28
225021_at	ZNF532	1,50E-28	1,10E-27
208960_s_at	KLF6	2,70E-27	1,90E-26
205255_x_at	TCF7	7,30E-27	5,00E-26
37254_at	ZNF133	9,90E-27	6,80E-26
218793_s_at	SCML1	2,00E-26	1,30E-25
1554411_at	CTNNB1	2,40E-25	1,60E-24
200990_at	TRIM28	3,10E-25	2,10E-24
208328_s_at	MEF2A	4,70E-25	3,10E-24
207443_at	NR2E1	1,20E-24	7,80E-24

206583_at	KRBOX4	3,30E-23	2,20E-22
209189_at	FOS	3,90E-23	2,50E-22
203985_at	ZNF212	4,30E-22	2,70E-21
217909_s_at	MLX	9,30E-22	5,90E-21
204057_at	IRF8	3,20E-21	2,00E-20
204275_at	CAPN15	2,70E-20	1,70E-19
1554465_s_at	KRBOX4	3,60E-20	2,20E-19
215933_s_at	HHEX	1,20E-19	7,20E-19
219548_at	ZNF16	2,90E-19	1,70E-18
204562_at	IRF4	6,00E-19	3,60E-18
236471_at	NFE2L3	1,20E-18	6,90E-18
224718_at	YY1	1,20E-18	6,90E-18
220055_at	ZNF287	1,80E-18	1,00E-17
201417_at	SOX4	2,50E-18	1,50E-17
218437_s_at	LZTFL1	1,20E-17	7,10E-17
236265_at	SP4	5,30E-17	3,00E-16
31845_at	ELF4	6,50E-17	3,60E-16
207001_x_at	TSC22D3	7,40E-17	4,10E-16
223216_x_at	ZNF395	1,20E-16	6,70E-16
205883_at	ZBTB16	2,20E-16	1,20E-15
242953_at	ZNF234	2,30E-16	1,30E-15
207920_x_at	ZFX	3,20E-16	1,70E-15
224806_at	TRIM25	4,00E-16	2,20E-15
210135_s_at	SHOX2	4,70E-16	2,50E-15
206931_at	ZNF141	6,10E-16	3,20E-15
204254_s_at	VDR	9,60E-16	5,00E-15
206219_s_at	VAV1	2,10E-15	1,10E-14
217294_s_at	ENO1	4,30E-15	2,20E-14
203585_at	ZNF185	1,30E-14	6,90E-14
200939_s_at	RERE	1,70E-14	8,50E-14
205619_s_at	MEOX1	2,30E-14	1,20E-13
201483_s_at	SUPT4H1	1,10E-13	5,40E-13
208270_s_at	RNPEP	2,00E-13	9,90E-13
200779_at	ATF4	3,90E-13	1,90E-12
219451_at	MSRB2	6,60E-13	3,20E-12
1554311_a_at	SUPT6H	1,20E-12	5,70E-12
201332_s_at	STAT6	2,80E-12	1,40E-11
202758_s_at	RFXANK	6,30E-12	3,00E-11
1565347_s_at	TFE3	7,10E-12	3,40E-11
210252_s_at	MADD	7,70E-12	3,60E-11
213452_at	ZNF184	1,40E-11	6,60E-11
218559_s_at	MAFB	1,40E-11	6,70E-11
218490_s_at	ZNF302	2,40E-11	1,10E-10
209454_s_at	TEAD3	2,90E-11	1,40E-10
201218_at	CTBP2	3,00E-11	1,40E-10
232555_at	CREB5	3,40E-11	1,50E-10
204206_at	MNT	3,40E-11	1,50E-10
217185_s_at	ZPR1	3,70E-10	1,70E-09
218724_s_at	TGIF2	4,90E-10	2,20E-09
218495_at	UXT	2,50E-09	1,10E-08
210818_s_at	BACH1	4,10E-09	1,80E-08
207002_s_at	PLAGL1	4,50E-09	2,00E-08
213639_s_at	ZNF500	1,00E-08	4,40E-08

235409_at	MGA	2,00E-08	8,40E-08
218215_s_at	NR1H2	2,00E-08	8,40E-08
218937_at	ZSCAN32	3,00E-08	1,30E-07
203543_s_at	KLF9	3,40E-08	1,40E-07
206053_at	ZNF510	4,50E-08	1,90E-07
210971_s_at	ARNTL	1,10E-07	4,40E-07
218221_at	ARNT	1,40E-07	6,00E-07
204234_s_at	ZNF195	1,60E-07	6,70E-07
205205_at	RELB	3,10E-07	1,30E-06
238761_at	ELK4	5,90E-07	2,40E-06
223819_x_at	COMMD5	1,00E-06	4,10E-06
213916_at	ZNF625-ZNF20	1,30E-06	5,40E-06
209239_at	NFKB1	1,80E-06	7,20E-06
206188_at	ZNF623	2,20E-06	8,90E-06
203643_at	ERF	2,50E-06	9,90E-06
218152_at	HMG20A	5,00E-06	2,00E-05
221645_s_at	ZNF83	6,70E-06	2,60E-05
217367_s_at	ZHX3	8,80E-06	3,40E-05
227067_x_at	NBPF14	1,30E-05	5,20E-05
212377_s_at	NOTCH2	2,20E-05	8,70E-05
218486_at	KLF11	2,40E-05	9,10E-05
1555325_s_at	ZNF26	3,70E-05	0,00014
203132_at	RB1	4,00E-05	0,00015
204798_at	MYB	4,10E-05	0,00015
229215_at	ASCL2	4,40E-05	0,00016
AFFX-HUMISGF3A/M97935_5_at	STAT1	6,90E-05	0,00026
202621_at	IRF3	7,30E-05	0,00027
232652_x_at	SCAND1	7,40E-05	0,00027
208056_s_at	CBFA2T3	0,00014	0,00051
200758_s_at	NFE2L1	0,00016	0,00058
203275_at	IRF2	0,0002	0,00072
200641_s_at	YWHAZP3	0,00021	0,00074
204481_at	BRPF1	0,00021	0,00075
202925_s_at	PLAGL2	0,00023	0,00082
231927_at	ATF6	0,00034	0,0012
229010_at	CBL	0,00053	0,0019
212618_at	ZNF609	0,00067	0,0024
205372_at	PLAG1	0,00088	0,0031
207513_s_at	ZNF189	0,00095	0,0033
219540_at	ZNF267	0,0011	0,0037
200052_s_at	ILF2	0,0013	0,0044
207980_s_at	CITED2	0,0014	0,0049
201507_at	PFDN1	0,0015	5
211097_s_at	PBX2	0,0018	6
225636_at	STAT2	0,0021	7
1553685_s_at	SP1	0,0022	0,0074
221873_at	ZNF143	0,0028	0,0095
228964_at	PRDM1	0,0029	0,0097
209088_s_at	UBN1	0,0037	12
233461_x_at	ZNF226	0,0052	17
213541_s_at	ERG	0,0085	28
50221_at	TFEB	0,0092	0,03
205089_at	ZNF7	11	37

203010_at	STAT5A	12	0,04
207338_s_at	ZNF200	14	44
201466_s_at	JUN	14	46
211965_at	ZFP36L1	15	46

Table S3

Affymetrix probe ID	Regulon	Pvalue	Adjusted.Pvalue
218767_at	REXO4	0	0
206142_at	ZNF135	5,70E-302	2,00E-299
200878_at	EPAS1	2,50E-260	5,80E-258
214482_at	ZBTB25	1,10E-218	1,80E-216
207704_s_at	GAS7	1,10E-213	1,50E-211
231940_at	ZNF529	1,30E-196	1,50E-194
209211_at	KLF5	1,50E-195	1,50E-193
203585_at	ZNF185	1,10E-187	9,40E-186
209930_s_at	NFE2	6,60E-187	5,10E-185
212501_at	CEBPB	1,80E-185	1,30E-183
205254_x_at	TCF7	2,30E-182	1,50E-180
209782_s_at	DBP	8,60E-171	5,00E-169
203394_s_at	HES1	5,70E-169	3,00E-167
214523_at	CEBPE	3,20E-162	1,60E-160
227150_at	MTF1	1,30E-158	5,90E-157
203574_at	NFIL3	2,60E-156	1,10E-154
204970_s_at	MAFG	1,70E-153	6,80E-152
230575_at	MSRB2	2,30E-151	8,70E-150
204327_s_at	ZNF202	4,20E-147	1,50E-145
214746_s_at	ZNF467	2,40E-146	8,40E-145
209602_s_at	GATA3	7,40E-144	2,40E-142
215285_s_at	PHTF1	1,80E-140	5,80E-139
205101_at	CIITA	2,00E-135	6,10E-134
207443_at	NR2E1	5,20E-132	1,50E-130
208510_s_at	PPARG	1,30E-131	3,60E-130
218284_at	SMAD3	1,10E-125	2,80E-124
205026_at	STAT5B	1,60E-123	4,10E-122
205883_at	ZBTB16	4,00E-117	9,90E-116
218184_at	TULP4	4,40E-113	1,00E-111
211721_s_at	ZNF551	8,50E-113	2,00E-111
228099_at	ZNF550	2,20E-109	4,70E-108
206118_at	STAT4	5,50E-107	1,20E-105
205312_at	SPI1	1,90E-106	3,90E-105
218880_at	FOSL2	1,30E-105	2,60E-104
209538_at	ZNF32	5,40E-103	1,00E-101
206965_at	KLF12	8,10E-102	1,50E-100
235567_at	RORA	6,90E-98	1,20E-96
205453_at	HOXB2	1,30E-95	2,30E-94
212892_at	ZNF282	2,50E-94	4,40E-93
216350_s_at	ZNF10	7,90E-93	1,30E-91
220055_at	ZNF287	4,70E-92	7,80E-91
213269_at	ZNF248	3,50E-90	5,70E-89
204432_at	SOX12	4,30E-90	6,80E-89
216305_s_at	GCFC2	4,80E-86	7,40E-85
205259_at	NR3C2	1,60E-84	2,40E-83
1558586_at	ZNF33B	2,70E-79	3,90E-78
206877_at	MXD1	1,30E-78	1,90E-77
219765_at	ZNF329	1,40E-74	2,00E-73
219548_at	ZNF16	3,00E-74	4,00E-73
201473_at	JUNB	4,50E-74	5,90E-73
200939_s_at	RERE	3,10E-73	4,00E-72
218437_s_at	LZTFL1	4,20E-73	5,40E-72

226872_at	RFX2	2,20E-72	2,70E-71
226102_at	ZKSCAN8	1,50E-70	1,80E-69
204562_at	IRF4	1,90E-69	2,20E-68
209200_at	MEF2C	3,80E-68	4,40E-67
242919_at	ZNF253	4,10E-68	4,60E-67
219228_at	ZNF331	5,00E-67	5,50E-66
201329_s_at	ETS2	2,90E-66	3,20E-65
209657_s_at	HSF2	4,90E-66	5,30E-65
206053_at	ZNF510	1,50E-64	1,60E-63
208808_s_at	HMGB2	2,30E-64	2,40E-63
205004_at	NKRF	2,60E-63	2,60E-62
201746_at	TP53	2,50E-62	2,60E-61
1555420_a_at	KLF7	9,30E-62	9,30E-61
220350_at	ZNF235	2,70E-59	2,70E-58
218735_s_at	ZNF544	4,80E-59	4,70E-58
236128_at	ZNF91	7,70E-58	7,40E-57
202963_at	RFX5	1,90E-55	1,80E-54
217627_at	ZNF573	2,30E-55	2,10E-54
208328_s_at	MEF2A	2,70E-52	2,50E-51
204039_at	CEBPA	6,10E-52	5,50E-51
220684_at	TBX21	9,80E-51	8,80E-50
202957_at	HCLS1	1,90E-49	1,70E-48
236471_at	NFE2L3	2,00E-48	1,80E-47
241365_at	SATB1	5,80E-45	5,00E-44
242953_at	ZNF234	2,50E-42	2,10E-41
215990_s_at	BCL6	4,60E-42	3,90E-41
203693_s_at	E2F3	1,20E-38	9,70E-38
217909_s_at	MLX	1,30E-38	1,00E-37
200989_at	HIF1A	2,00E-37	1,60E-36
1554411_at	CTNNB1	6,50E-37	5,00E-36
201483_s_at	SUPT4H1	1,10E-36	8,40E-36
210062_s_at	ZNF589	1,70E-36	1,30E-35
227132_at	ZNF706	7,30E-36	5,50E-35
211097_s_at	PBX2	1,10E-35	8,40E-35
1554465_s_at	KRBOX4	7,30E-34	5,40E-33
218149_s_at	ZNF395	7,30E-33	5,30E-32
219854_at	ZNF14	1,70E-32	1,20E-31
227045_at	ZNF614	8,90E-31	6,30E-30
204255_s_at	VDR	1,70E-30	1,20E-29
207128_s_at	ZNF223	2,00E-30	1,40E-29
228466_at	GABPB2	2,50E-30	1,70E-29
229431_at	RFXAP	6,10E-30	4,10E-29
209189_at	FOS	5,20E-29	3,50E-28
229228_at	CREB5	7,50E-29	5,00E-28
206182_at	ZNF134	8,20E-29	5,30E-28
225021_at	ZNF532	9,80E-28	6,30E-27
1555355_a_at	ETS1	1,40E-27	8,80E-27
227198_at	AFF3	2,30E-27	1,40E-26
205865_at	ARID3A	8,10E-27	5,00E-26
211789_s_at	MLXIP	2,20E-26	1,40E-25
209798_at	NPAT	6,30E-26	3,80E-25
201507_at	PFDN1	2,00E-25	1,20E-24
218006_s_at	ZNF22	2,20E-24	1,30E-23

204194_at	BACH1	1,70E-23	9,70E-23
217185_s_at	ZPR1	2,10E-23	1,20E-22
218559_s_at	MAFB	8,30E-23	4,80E-22
218724_s_at	TGIF2	1,10E-22	6,40E-22
208270_s_at	RNPEP	3,20E-22	1,80E-21
215933_s_at	HHEX	6,60E-22	3,60E-21
208960_s_at	KLF6	4,60E-21	2,50E-20
212079_s_at	KMT2A	1,60E-20	8,50E-20
200050_at	ZNF146	1,30E-19	7,10E-19
224718_at	YY1	1,40E-19	7,40E-19
224806_at	TRIM25	3,50E-19	1,80E-18
235122_at	HIVEP3	4,00E-19	2,10E-18
218215_s_at	NR1H2	4,30E-19	2,20E-18
205619_s_at	MEOX1	5,90E-19	3,00E-18
205205_at	RELB	3,50E-18	1,80E-17
202758_s_at	RFXANK	5,70E-18	2,90E-17
204791_at	NR2C1	1,30E-17	6,40E-17
37254_at	ZNF133	1,10E-16	5,60E-16
1554311_a_at	SUPT6H	2,70E-16	1,30E-15
204045_at	TCEAL3	7,30E-15	3,40E-14
206219_s_at	VAV1	1,50E-14	6,90E-14
218793_s_at	SCML1	6,30E-14	2,90E-13
207753_at	ZNF304	1,00E-13	4,70E-13
201332_s_at	STAT6	1,50E-13	7,00E-13
236265_at	SP4	4,00E-13	1,80E-12
204057_at	IRF8	1,40E-12	6,40E-12
200758_s_at	NFE2L1	1,60E-12	7,30E-12
201417_at	SOX4	1,80E-12	8,10E-12
1555325_s_at	ZNF26	5,30E-12	2,30E-11
232652_x_at	SCAND1	1,90E-10	8,20E-10
218401_s_at	ZNF281	2,80E-10	1,20E-09
1565347_s_at	TFE3	4,20E-10	1,80E-09
217294_s_at	ENO1	6,00E-10	2,50E-09
201218_at	CTBP2	2,20E-09	9,20E-09
218490_s_at	ZNF302	3,00E-09	1,20E-08
206931_at	ZNF141	3,10E-09	1,30E-08
213541_s_at	ERG	1,10E-08	4,60E-08
207980_s_at	CITED2	2,40E-08	9,50E-08
207001_x_at	TSC22D3	3,90E-08	1,60E-07
229010_at	CBL	5,60E-08	2,20E-07
201466_s_at	JUN	6,40E-08	2,50E-07
205170_at	STAT2	1,10E-07	4,30E-07
203275_at	IRF2	1,70E-07	6,70E-07
210252_s_at	MADD	4,50E-07	1,70E-06
200990_at	TRIM28	1,40E-06	5,20E-06
218486_at	KLF11	1,60E-06	5,90E-06
31845_at	ELF4	3,30E-06	1,20E-05
209454_s_at	TEAD3	3,50E-06	1,30E-05
204275_at	CAPN15	7,00E-06	2,60E-05
50221_at	TFEB	7,40E-06	2,70E-05
227067_x_at	NBPF14	1,10E-05	3,80E-05
213452_at	ZNF184	1,10E-05	4,00E-05
203985_at	ZNF212	5,00E-05	0,00018

218152_at	HMG20A	5,10E-05	0,00018
207513_s_at	ZNF189	0,00016	0,00054
204798_at	MYB	0,0002	0,0007
210756_s_at	NOTCH2	0,00026	0,00089
219540_at	ZNF267	0,00028	0,00094
203543_s_at	KLF9	0,00032	0,0011
205372_at	PLAG1	0,00037	0,0012
233461_x_at	ZNF226	0,00045	0,0015
AFFX-HUMISGF3A/M97935_5_at	STAT1	0,00047	0,0015
207002_s_at	PLAGL1	0,00087	0,0029
208056_s_at	CBFA2T3	0,0015	0,0047
213639_s_at	ZNF500	0,0023	0,0073
218495_at	UXT	0,0036	11
204481_at	BRPF1	0,0037	12
200641_s_at	YWHAZP3	4	12
208443_x_at	SHOX2	0,0056	18
231927_at	ATF6	0,0068	21
213916_at	ZNF625-ZNF20	7	22
203132_at	RB1	0,0072	22
202925_s_at	PLAGL2	0,0082	25
209088_s_at	UBN1	0,01	0,03

Table S4

Affymetrix probe ID	Regulon	Pvalue	Adjusted.Pvalue
214482_at	ZBTB25	1,10E-237	7,90E-235
206142_at	ZNF135	1,40E-197	4,70E-195
231940_at	ZNF529	1,60E-177	3,60E-175
209930_s_at	NFE2	2,10E-167	3,60E-165
211721_s_at	ZNF551	6,80E-164	9,40E-162
204327_s_at	ZNF202	7,90E-161	9,10E-159
209602_s_at	GATA3	1,40E-154	1,40E-152
204970_s_at	MAFG	2,30E-154	2,00E-152
200878_at	EPAS1	2,60E-150	2,00E-148
227150_at	MTF1	5,00E-147	3,40E-145
213269_at	ZNF248	4,90E-138	3,10E-136
207704_s_at	GAS7	9,30E-135	5,30E-133
203574_at	NFIL3	2,60E-133	1,40E-131
212501_at	CEBPB	3,10E-120	1,50E-118
214523_at	CEBPE	1,60E-115	7,40E-114
206965_at	KLF12	4,20E-115	1,80E-113
209211_at	KLF5	6,00E-114	2,40E-112
204432_at	SOX12	3,70E-113	1,40E-111
1558586_at	ZNF33B	1,40E-107	5,10E-106
215285_s_at	PHTF1	2,40E-103	8,20E-102
226682_at	RORA	1,50E-102	5,00E-101
1555420_a_at	KLF7	2,40E-102	7,60E-101
214746_s_at	ZNF467	5,40E-102	1,60E-100
226102_at	ZKSCAN8	6,40E-101	1,80E-99
218184_at	TULP4	1,90E-95	5,10E-94
205026_at	STAT5B	1,50E-90	4,00E-89
218767_at	REXO4	4,20E-89	1,10E-87
208510_s_at	PPARG	1,80E-88	4,50E-87
209782_s_at	DBP	8,20E-87	1,90E-85
219765_at	ZNF329	3,10E-84	7,10E-83
205004_at	NKRF	1,40E-82	3,00E-81
217627_at	ZNF573	9,40E-78	2,00E-76
242919_at	ZNF253	1,00E-76	2,10E-75
220350_at	ZNF235	5,20E-76	1,10E-74
216305_s_at	GCFC2	2,80E-74	5,50E-73
218880_at	FOSL2	1,10E-69	2,00E-68
202963_at	RFX5	9,00E-69	1,70E-67
220684_at	TBX21	9,30E-69	1,70E-67
201329_s_at	ETS2	2,60E-65	4,60E-64
228099_at	ZNF550	2,70E-65	4,60E-64
218284_at	SMAD3	6,70E-64	1,10E-62
206877_at	MXD1	1,00E-62	1,70E-61
209538_at	ZNF32	2,40E-62	3,80E-61
215990_s_at	BCL6	8,70E-62	1,40E-60
201746_at	TP53	3,00E-59	4,60E-58
205453_at	HOXB2	7,90E-59	1,20E-57
212079_s_at	KMT2A	1,60E-58	2,30E-57
219228_at	ZNF331	4,10E-56	5,70E-55
205312_at	SPI1	1,00E-55	1,40E-54
241365_at	SATB1	8,80E-55	1,20E-53
204791_at	NR2C1	1,30E-50	1,70E-49
227132_at	ZNF706	1,10E-49	1,40E-48

206118_at	STAT4	1,30E-49	1,60E-48
228466_at	GABPB2	1,40E-49	1,70E-48
229431_at	RFXAP	2,90E-48	3,60E-47
205101_at	CIITA	3,70E-48	4,30E-47
226872_at	RFX2	7,70E-47	9,00E-46
218149_s_at	ZNF395	1,60E-46	1,80E-45
205865_at	ARID3A	1,70E-46	1,90E-45
209657_s_at	HSF2	9,00E-45	9,80E-44
209200_at	MEF2C	7,10E-44	7,50E-43
203693_s_at	E2F3	1,90E-41	2,00E-40
229228_at	CREB5	2,20E-41	2,20E-40
204194_at	BACH1	3,10E-41	3,10E-40
204039_at	CEBPA	1,80E-40	1,80E-39
211789_s_at	MLXIP	5,40E-40	5,30E-39
201473_at	JUNB	1,30E-38	1,30E-37
207753_at	ZNF304	1,70E-38	1,70E-37
210062_s_at	ZNF589	1,60E-37	1,50E-36
218735_s_at	ZNF544	5,50E-37	5,00E-36
207128_s_at	ZNF223	9,30E-37	8,40E-36
219854_at	ZNF14	1,10E-36	1,00E-35
227045_at	ZNF614	1,20E-36	1,00E-35
202957_at	HCLS1	4,70E-36	4,10E-35
235122_at	HIVEP3	5,00E-36	4,30E-35
227198_at	AFF3	2,90E-35	2,50E-34
203394_s_at	HES1	1,20E-33	1,00E-32
205259_at	NR3C2	1,40E-33	1,20E-32
216350_s_at	ZNF10	1,50E-33	1,20E-32
1555355_a_at	ETS1	2,10E-33	1,70E-32
218006_s_at	ZNF22	6,70E-33	5,30E-32
209798_at	NPAT	1,20E-32	9,40E-32
208808_s_at	HMGB2	1,60E-31	1,20E-30
200989_at	HIF1A	2,80E-31	2,10E-30
204045_at	TCEAL3	8,60E-31	6,50E-30
236128_at	ZNF91	1,60E-30	1,20E-29
212892_at	ZNF282	1,70E-30	1,30E-29
206182_at	ZNF134	1,80E-30	1,30E-29
200050_at	ZNF146	1,40E-29	1,00E-28
218401_s_at	ZNF281	1,20E-28	8,30E-28
225021_at	ZNF532	1,50E-28	1,10E-27
208960_s_at	KLF6	2,70E-27	1,90E-26
205255_x_at	TCF7	7,30E-27	5,00E-26
37254_at	ZNF133	9,90E-27	6,80E-26
218793_s_at	SCML1	2,00E-26	1,30E-25
1554411_at	CTNNB1	2,40E-25	1,60E-24
200990_at	TRIM28	3,10E-25	2,10E-24
208328_s_at	MEF2A	4,70E-25	3,10E-24
207443_at	NR2E1	1,20E-24	7,80E-24
206583_at	KRBOX4	3,30E-23	2,20E-22
209189_at	FOS	3,90E-23	2,50E-22
203985_at	ZNF212	4,30E-22	2,70E-21
217909_s_at	MLX	9,30E-22	5,90E-21
204057_at	IRF8	3,20E-21	2,00E-20
204275_at	CAPN15	2,70E-20	1,70E-19

215933_s_at	HHEX	1,20E-19	7,20E-19
219548_at	ZNF16	2,90E-19	1,70E-18
204562_at	IRF4	6,00E-19	3,60E-18
236471_at	NFE2L3	1,20E-18	6,90E-18
224718_at	YY1	1,20E-18	6,90E-18
220055_at	ZNF287	1,80E-18	1,00E-17
201417_at	SOX4	2,50E-18	1,50E-17
218437_s_at	LZTFL1	1,20E-17	7,10E-17
236265_at	SP4	5,30E-17	3,00E-16
31845_at	ELF4	6,50E-17	3,60E-16
207001_x_at	TSC22D3	7,40E-17	4,10E-16
205883_at	ZBTB16	2,20E-16	1,20E-15
242953_at	ZNF234	2,30E-16	1,30E-15
224806_at	TRIM25	4,00E-16	2,20E-15
210135_s_at	SHOX2	4,70E-16	2,50E-15
206931_at	ZNF141	6,10E-16	3,20E-15
204254_s_at	VDR	9,60E-16	5,00E-15
206219_s_at	VAV1	2,10E-15	1,10E-14
217294_s_at	ENO1	4,30E-15	2,20E-14
203585_at	ZNF185	1,30E-14	6,90E-14
200939_s_at	RERE	1,70E-14	8,50E-14
205619_s_at	MEOX1	2,30E-14	1,20E-13
201483_s_at	SUPT4H1	1,10E-13	5,40E-13
208270_s_at	RNPEP	2,00E-13	9,90E-13
219451_at	MSRB2	6,60E-13	3,20E-12
1554311_a_at	SUPT6H	1,20E-12	5,70E-12
201332_s_at	STAT6	2,80E-12	1,40E-11
202758_s_at	RFXANK	6,30E-12	3,00E-11
1565347_s_at	TFE3	7,10E-12	3,40E-11
210252_s_at	MADD	7,70E-12	3,60E-11
213452_at	ZNF184	1,40E-11	6,60E-11
218559_s_at	MAFB	1,40E-11	6,70E-11
218490_s_at	ZNF302	2,40E-11	1,10E-10
209454_s_at	TEAD3	2,90E-11	1,40E-10
201218_at	CTBP2	3,00E-11	1,40E-10
217185_s_at	ZPR1	3,70E-10	1,70E-09
218724_s_at	TGIF2	4,90E-10	2,20E-09
218495_at	UXT	2,50E-09	1,10E-08
207002_s_at	PLAGL1	4,50E-09	2,00E-08
213639_s_at	ZNF500	1,00E-08	4,40E-08
218215_s_at	NR1H2	2,00E-08	8,40E-08
203543_s_at	KLF9	3,40E-08	1,40E-07
206053_at	ZNF510	4,50E-08	1,90E-07
205205_at	RELB	3,10E-07	1,30E-06
213916_at	ZNF625-ZNF20	1,30E-06	5,40E-06
218152_at	HMG20A	5,00E-06	2,00E-05
227067_x_at	NBPF14	1,30E-05	5,20E-05
212377_s_at	NOTCH2	2,20E-05	8,70E-05
218486_at	KLF11	2,40E-05	9,10E-05
1555325_s_at	ZNF26	3,70E-05	0,00014
203132_at	RB1	4,00E-05	0,00015
204798_at	MYB	4,10E-05	0,00015
AFFX-HUMISGF3A/M97935_5_at	STAT1	6,90E-05	0,00026

232652_x_at	SCAND1	7,40E-05	0,00027
208056_s_at	CBFA2T3	0,00014	0,00051
200758_s_at	NFE2L1	0,00016	0,00058
203275_at	IRF2	0,0002	0,00072
200641_s_at	YWHAZP3	0,00021	0,00074
204481_at	BRPF1	0,00021	0,00075
202925_s_at	PLAGL2	0,00023	0,00082
231927_at	ATF6	0,00034	0,0012
229010_at	CBL	0,00053	0,0019
205372_at	PLAG1	0,00088	0,0031
207513_s_at	ZNF189	0,00095	0,0033
219540_at	ZNF267	0,0011	0,0037
207980_s_at	CITED2	0,0014	0,0049
201507_at	PFDN1	0,0015	5
211097_s_at	PBX2	0,0018	6
225636_at	STAT2	0,0021	7
209088_s_at	UBN1	0,0037	12
233461_x_at	ZNF226	0,0052	17
213541_s_at	ERG	0,0085	28
50221_at	TFEB	0,0092	0,03
201466_s_at	JUN	14	46

Table S5

Affymetrix probe ID	Regulon	Pvalue	Adjusted.Pvalue	Cluster
218767_at	REXO4	0	0	B
200878_at	EPAS1	2,50E-260	5,80E-258	B
214482_at	ZBTB25	1,10E-218	1,80E-216	B
207704_s_at	GAS7	1,10E-213	1,50E-211	B
231940_at	ZNF529	1,30E-196	1,50E-194	A
209211_at	KLF5	1,50E-195	1,50E-193	B
212501_at	CEBPB	1,80E-185	1,30E-183	B
209782_s_at	DBP	8,60E-171	5,00E-169	B
203394_s_at	HES1	5,70E-169	3,00E-167	B
227150_at	MTF1	1,30E-158	5,90E-157	B
203574_at	NFIL3	2,60E-156	1,10E-154	B
204970_s_at	MAFG	1,70E-153	6,80E-152	B
204327_s_at	ZNF202	4,20E-147	1,50E-145	A
214746_s_at	ZNF467	2,40E-146	8,40E-145	B
209602_s_at	GATA3	7,40E-144	2,40E-142	A
215285_s_at	PHTF1	1,80E-140	5,80E-139	B
205101_at	CIITA	2,00E-135	6,10E-134	B
218284_at	SMAD3	1,10E-125	2,80E-124	A
218184_at	TULP4	4,40E-113	1,00E-111	A
211721_s_at	ZNF551	8,50E-113	2,00E-111	A
218880_at	FOSL2	1,30E-105	2,60E-104	B
206965_at	KLF12	8,10E-102	1,50E-100	A
235567_at	RORA	6,90E-98	1,20E-96	A
205453_at	HOXB2	1,30E-95	2,30E-94	A
216350_s_at	ZNF10	7,90E-93	1,30E-91	B
204432_at	SOX12	4,30E-90	6,80E-89	B
216305_s_at	GCFC2	4,80E-86	7,40E-85	A
205259_at	NR3C2	1,60E-84	2,40E-83	A
219765_at	ZNF329	1,40E-74	2,00E-73	A
219548_at	ZNF16	3,00E-74	4,00E-73	B
201473_at	JUNB	4,50E-74	5,90E-73	B
226872_at	RFX2	2,20E-72	2,70E-71	B
226102_at	ZKSCAN8	1,50E-70	1,80E-69	A
204562_at	IRF4	1,90E-69	2,20E-68	A
209200_at	MEF2C	3,80E-68	4,40E-67	A
242919_at	ZNF253	4,10E-68	4,60E-67	A
219228_at	ZNF331	5,00E-67	5,50E-66	A
206053_at	ZNF510	1,50E-64	1,60E-63	B
205004_at	NKRF	2,60E-63	2,60E-62	A
1555420_a_at	KLF7	9,30E-62	9,30E-61	B
220350_at	ZNF235	2,70E-59	2,70E-58	A
218735_s_at	ZNF544	4,80E-59	4,70E-58	A
208328_s_at	MEF2A	2,70E-52	2,50E-51	B
236471_at	NFE2L3	2,00E-48	1,80E-47	B
242953_at	ZNF234	2,50E-42	2,10E-41	A
215990_s_at	BCL6	4,60E-42	3,90E-41	B
227132_at	ZNF706	7,30E-36	5,50E-35	A
206182_at	ZNF134	8,20E-29	5,30E-28	B
224806_at	TRIM25	3,50E-19	1,80E-18	B
206931_at	ZNF141	3,10E-09	1,30E-08	A

Table S6

Affymetrix probe ID	Regulon	Pvalue	Adjusted.Pvalue	Cluster
214482_at	ZBTB25	1,10E-237	7,90E-235	B
231940_at	ZNF529	1,60E-177	3,60E-175	A
211721_s_at	ZNF551	6,80E-164	9,40E-162	A
204327_s_at	ZNF202	7,90E-161	9,10E-159	A
209602_s_at	GATA3	1,40E-154	1,40E-152	A
204970_s_at	MAFG	2,30E-154	2,00E-152	B
200878_at	EPAS1	2,60E-150	2,00E-148	B
227150_at	MTF1	5,00E-147	3,40E-145	B
207704_s_at	GAS7	9,30E-135	5,30E-133	B
203574_at	NFIL3	2,60E-133	1,40E-131	B
212501_at	CEBPB	3,10E-120	1,50E-118	B
206965_at	KLF12	4,20E-115	1,80E-113	A
209211_at	KLF5	6,00E-114	2,40E-112	B
204432_at	SOX12	3,70E-113	1,40E-111	B
215285_s_at	PHTF1	2,40E-103	8,20E-102	B
226682_at	RORA	1,50E-102	5,00E-101	A
1555420_a_at	KLF7	2,40E-102	7,60E-101	B
214746_s_at	ZNF467	5,40E-102	1,60E-100	B
226102_at	ZKSCAN8	6,40E-101	1,80E-99	A
218184_at	TULP4	1,90E-95	5,10E-94	A
218767_at	REXO4	4,20E-89	1,10E-87	B
209782_s_at	DBP	8,20E-87	1,90E-85	B
219765_at	ZNF329	3,10E-84	7,10E-83	A
205004_at	NKRF	1,40E-82	3,00E-81	A
242919_at	ZNF253	1,00E-76	2,10E-75	A
220350_at	ZNF235	5,20E-76	1,10E-74	A
216305_s_at	GCFC2	2,80E-74	5,50E-73	A
218880_at	FOSL2	1,10E-69	2,00E-68	B
218284_at	SMAD3	6,70E-64	1,10E-62	A
215990_s_at	BCL6	8,70E-62	1,40E-60	B
205453_at	HOXB2	7,90E-59	1,20E-57	A
219228_at	ZNF331	4,10E-56	5,70E-55	A
227132_at	ZNF706	1,10E-49	1,40E-48	A
205101_at	CIITA	3,70E-48	4,30E-47	A
226872_at	RFX2	7,70E-47	9,00E-46	B
209200_at	MEF2C	7,10E-44	7,50E-43	A
201473_at	JUNB	1,30E-38	1,30E-37	B
218735_s_at	ZNF544	5,50E-37	5,00E-36	A
203394_s_at	HES1	1,20E-33	1,00E-32	B
205259_at	NR3C2	1,40E-33	1,20E-32	A
216350_s_at	ZNF10	1,50E-33	1,20E-32	B
206182_at	ZNF134	1,80E-30	1,30E-29	A
208328_s_at	MEF2A	4,70E-25	3,10E-24	B
219548_at	ZNF16	2,90E-19	1,70E-18	B
204562_at	IRF4	6,00E-19	3,60E-18	A
236471_at	NFE2L3	1,20E-18	6,90E-18	B
242953_at	ZNF234	2,30E-16	1,30E-15	A
224806_at	TRIM25	4,00E-16	2,20E-15	B
206931_at	ZNF141	6,10E-16	3,20E-15	A
206053_at	ZNF510	4,50E-08	1,90E-07	B

Table S7

Regulon	Pvalue	Adjusted.Pvalue
ZNF529	1.3e-196	1.5e-194
GATA3	7.4e-144	2.4e-142
KLF12	8.1e-102	1.5e-100
RORA	6.9e-98	1.2e-96
HOXB2	1.3e-95	2.3e-94
NR3C2	1.6e-84	2.4e-83
ZNF329	1.4e-74	2.0e-073
RFX2	2.2e-72	2.7e-71
ZKSCAN8	1.5e-70	1.8e-69
ZNF331	5.0e-067	5.5e-66
ZNF235	2.7e-59	2.7e-58
MEF2A	2.7e-52	2.5e-51
ZNF234	2.5e-42	2.1e-41
ZNF134	8.2e-29	5.3e-28
TRIM25	3.5e-19	1.8e-18

Table S8

Regulon	Pvalue	Adjusted.Pvalue
ZNF529	1.6e-177	3.6e-175
GATA3	1.4e-154	1.4e-152
KLF12	4.2e-115	1.8e-113
RORA	1.5e-102	5.0e-101
ZKSCAN8	6.4e-101	1.8e-99
ZNF329	3.1e-84	7.1e-83
ZNF235	5.2e-76	1.1e-74
HOXB2	7.9e-59	1.2e-57
ZNF331	4.1e-56	5.7e-55
RFX2	7.7e-47	9.0e-046
NR3C2	1.4e-33	1.2e-32
ZNF134	1.8e-30	1.3e-29
MEF2A	4.7e-25	3.1e-24
ZNF234	2.3e-16	1.3e-15
TRIM25	4,00E-16	2.2e-15

Table S9

Gene	Other aliases	Protein
**ZNF529**	Zinc Finger Protein 529,<br>KIAA1615.	Zinc finger protein 529
**GATA3**	GATA Binding Protein,<br>Trans-Acting T-Cell-Specific Transcription Factor GATA-3,<br>GATA-Binding Factor,<br>HDR,<br>GATA-Binding Protein,<br>HDRS.	Trans-acting T-cell-specific transcription factor GATA-3
**KLF12**	Kruppel Like Factor 12,<br>AP2REP,<br>KLF12 Zinc Finger Transcriptional Repressor,<br>Transcriptional Repressor AP-2rep,<br>AP-2rep Transcription Factor.	Krueppel-like factor 12
**RORA**	RAR Related Orphan Receptor A,<br>NR1F1,<br>RZRA,<br>Nuclear Receptor Subfamily 1 Group F Member 1,<br>RAR-Related Orphan Receptor A,<br>Nuclear Receptor ROR-Alpha,<br>Nuclear Receptor RZR-Alpha,<br>ROR1,<br>ROR2,<br>ROR3,<br>Retinoic Acid Receptor-Related Orphan Receptor Alpha,<br>Thyroid Hormone Nuclear Receptor Alpha Variant 4,<br>Retinoid-Related Orphan Receptor Alpha,<br>Retinoid-Related Orphan Receptor-Alpha<br>Transcription Factor RZR-Alpha,<br>RZR-ALPHA,<br>ROR-Alpha,<br>IDDECA,<br>RORA.	Nuclear receptor ROR-alpha
**HOXB2**	Homeobox B2,<br>Homeobox Protein Hox-2.8,<br>Homeobox Protein Hox-B2,<br>Homeobox Protein Hox-2H,<br>Homeo Box B2,<br>HOX2H,<br>K8,<br>K8 Home Protein,<br>Homeo Box 2H,<br>Hox-2.8,<br>HOXB2,<br>HOX2.	Homeobox protein Hox-B2
**NR3C2**	Nuclear Receptor Subfamily 3 Group C Member 2,<br>MR,<br>Mineralocorticoid Receptor,<br>MCR,<br>MLR,<br>Nuclear Receptor Subfamily 3, Group C, Member 2 Variant 3,<br>Nuclear Receptor Subfamily 3, Group C, Member 2,<br>Mineralocorticoid Receptor Delta,<br>Mineralocorticoid Receptor 1,<br>Mineralocorticoid Receptor 2,<br>Aldosterone Receptor,<br>NR3C2VIT,<br>NR3C2.	Mineralocorticoid receptor

**ZNF329**	Zinc Finger Protein 329,<br>FLJ12586,<br>ZNF329.	Zinc finger protein 329
**RFX2**	Regulatory Factor X2,<br>Regulatory Factor X, 2 (Influences HLA Class II Expression),<br>HLA Class II Regulatory Factor RFX2,<br>Trans-Acting Regulatory Factor 2,<br>DNA-Binding Protein RFX2,<br>Regulatory Factor X 2,<br>DNA Binding Protein RFX2,<br>FLJ14226,<br>RFX2.	DNA-binding protein RFX2
**ZKSCAN8**	Zinc Finger With KRAB And SCAN Domains 8,<br>Zinc Finger Protein 192,<br>LD5-1,<br>Zinc Finger Protein With KRAB And SCAN Domains 8,<br>ZSCAN40,<br>ZNF192.	Zinc finger protein with KRAB and SCAN domains 8
**ZNF331**	Zinc Finger Protein 331,<br>ZNF361,<br>ZNF463,<br>RITA,<br>C2H2-Like Zinc Finger Protein Rearranged In Thyroid Adenomas,<br>Zinc Finger Protein 361,<br>Zinc Finger Protein 463,<br>Rearranged In Thyroid Adenomas,<br>KRAB Zinc Finger Protein.	Zinc finger protein 331
**ZNF235**	Zinc Finger Protein 235,<br>Zinc Finger Protein Homologous To Zfp93 In Mouse,<br>Zinc Finger Protein 93 Homolog,<br>Zinc Finger Protein HZF6,<br>Zinc Finger Protein 270,<br>ANF270,<br>ZNF270,<br>Zfp-93,<br>ZFP93,<br>HZF6,<br>Zinc Finger Protein Homologous To Mouse Zfp93,<br>Zinc Finger Protein 6.	Zinc finger protein 235
**MEF2A**	Myocyte Enhancer Factor 2A,<br>Serum Response Factor-Like Protein 1,<br>Myocyte-Specific Enhancer Factor 2A,<br>RSRFC4,<br>RSRFC9,<br>MADS Box Transcription Enhancer Factor 2, Polypeptide A (Myocyte Enhancer Factor 2A),<br>ADCAD1,<br>MEF2A,<br>Mef2,<br>MEF2.	Myocyte-specific enhancer factor 2A
**ZNF234**	Zinc Finger Protein 234,<br>Zinc Finger Protein HZF4,<br>Zinc Finger Protein 269,<br>ZNF269,<br>HZF4,<br>Zinc Finger Protein 234, Partial Sequence,<br>C2-H2 Type Zinc Finger Protein,<br>Homo Sapiens Zinc Finger 234,<br>Zinc Finger Protein.	Zinc finger protein 234

Table S9

**ZNF134**	Zinc Finger Protein 134,<br>Zinc Finger Protein 134 (Clone PHZ-15).<br>PHZ-15.	Zinc finger protein 134
**TRIM25**	Tripartite Motif Containing 25,<br>RNF147,<br>EFP,<br>Zinc Finger Protein 147 (Estrogen-Responsive Finger Protein),<br>RING-Type E3 Ubiquitin Transferase TRIM25,<br>Ubiquitin/ISG15-Conjugating Enzyme TRIM25,<br>Tripartite Motif-Containing Protein 25,<br>Estrogen-Responsive Finger Protein,<br>E3 Ubiquitin/ISG15 Ligase TRIM25,<br>RING Finger Protein 147,<br>ZNF147,<br>RING-Type E3 Ubiquitin Transferase,<br>Tripartite Motif Protein TRIM25,<br>Tripartite Motif-Containing 25,<br>Zinc Finger Protein-147,<br>Zinc Finger Protein 147,<br>EC 6.3.2.n3,<br>EC 2.3.2.27,<br>Z147.	E3 ubiquitin/ISG15 ligase TRIM25

Information obtained on GeneCards.
