# Supplementary figures and images for: Reverse Engineering of the Pediatric Sepsis Regulatory Network and Identification of Master Regulators
Source: Biomedicines. 2021 Sep 23;9(10):1297. doi: 10.3390/biomedicines9101297 (PMC8533457; doi:10.3390/biomedicines9101297)

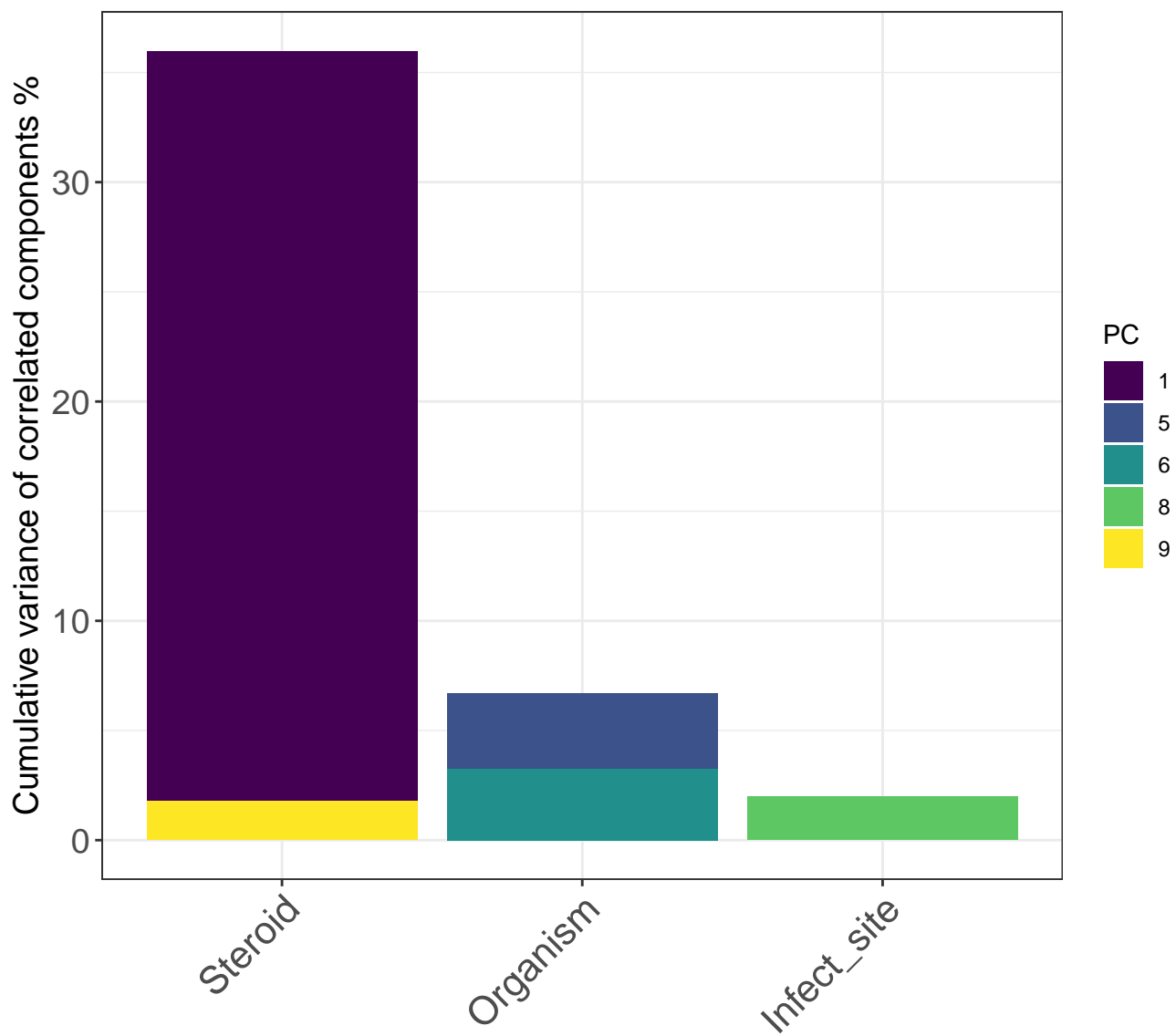

Supplement: Supplementary file 1 [file biomedicines-09-01297-s001.zip › Supplementary Figure S3.pdf]
